# The prevalence of post-traumatic stress disorder among emergency nurses: a cross sectional study in northern Iran

**DOI:** 10.1051/bmdcn/2019090319

**Published:** 2019-08-27

**Authors:** Seyed Mohammad Hosseininejad, Fatemeh Jahanian, Forouzan Elyasi, Hossein Mokhtari, Mohammad Eslami Koulaei, Seyedeh Masoumeh Pashaei

**Affiliations:** 1 Department of Emergency Medicine, Diabetes Research Center, Mazandaran University of Medical Sciences Sari Iran; 2 Department of Emergency Medicine, Gut and Liver Research Center, Mazandaran University of Medical Sciences Sari Iran; 3 Psychiatry and Behavioral Sciences Research Center, Addiction Institute, Faculty of Medicine, Mazandaran University of Medical Science Sari Iran; 4 Head Nurse of Emergency Department, Sari Imam Khomeini Hospital, Mazandaran University of Medical Science Sari Iran; 5 Department of Emergency Medicine, Faculty of Medicine, Mazandaran University of Medical Sciences Sari Iran; 6 Department of Emergency Medicine, Orthopedic Research Center, Mazandaran University of Medical Sciences Sari Iran

**Keywords:** Stress Disorders, Post-Traumatic, Nurses, Emergency Service, Hospital

## Abstract

Background and objective: Posttraumatic stress disorder (PTSD) is one of the anxiety disorders which occurs in response to facing events and accidents accompanied by fear, frustration, and terror. Nurses who worked in the emergency departments witness unpleasant accidents and are exposed to stress and violence more than others. The aim of this study was to investigate the prevalence of PTSD among Iranian nurses working in the emergency department.

Materials and Methods: In this cross-sectional study, all nurses working in the emergency department of educational hospitals affiliated with Mazandaran University of Medical Sciences were included through census method (n = 131). Each of the participants in this study responded to the questionnaires individually. The first part of the questionnaire captured demographics, while the second part was the Civilian Mississippi Scale for PTSD, which was used to investigate the extent of PTSD in this study. Data were analyzed using SPSS version 22.

Results: In this study, 131 respondents were included. In the classification of age, nurses with 20-30-year- old had the maximum frequency (41.2 %,). Most nurses in the study (93.1%, n = 122) had a bachelor’s degree in nursing and 84 were married. The prevalence of PTSD in emergency nurses was 82.96%, which was higher in nurses with shorter working background and nurses with lower levels of education. Further, the average score of PTSD was higher in married nurses.

Conclusion: The results of this study revealed the high prevalence of PTSD among nurses who worked in emergency department. Therefore, it seems that designing and providing supportive and educational services to emergency nurses can be effective for preventing and managing this disorder, which probably can improve their performance.

## 1. Introduction

Posttraumatic stress disorder (PTSD) is an anxiety disorder which occurs in response to experiencing accidents and events accompanied by fear, frustration, and terror [1–4]. The main symptoms of PTSD are intrusive and unwanted images and memories of accidents and behavioral avoidance from the signs that remind a person of those events. This disorder impairs normal occupational functioning of the affected person [5, 6]. Initially, PTSD was identified in those who had recently been traumatized (such as soldiers returning from war, victims of rape, kidnapping, and hostage) [7–9]. Nevertheless, subsequent studies indicated that Khomeini Hospital, Amir Mazandarani Street, Sari, Mazandaran, PTSD can also develop in those who witness disastrous events in the workplace [10–15]. Stress can be defined as a bio-psychological reaction of the body to physical and mental conditions that threaten the health or life of the person. Previous studies suggest that living with stress can contribute to developing PTSD [5, 12, 16].

Considering the special conditions and complexity of hospitals, which are associated with daily unpredictable working conditions, difficult tasks, unrealistic requests and expectations of patients and their companions, and taking care of emergency and terminally ill patients and witnessing the death of patients, create highly stressful conditions for nurses who are directly in contact with patients for prolonged periods. This can cause development of job-related psychological problems such as PTSD symptoms [17–19]. Around one thirds of nurses in the emergency units have PTSD symptoms, out of them, 12-18% fulfill all of the conditions for diagnosis of PTSD. In a study conducted in the US, PTSD symptoms were reported in the nurses of intensive care units and emergency units in 20-30% of cases, which is significantly different in comparison to the general population of the US, with a prevalence of 3.5% [20–24].

One of the most important indicators of the efficiency of the healthcare system is the satisfaction of patients with hospital services. One of the most essential parts of it is related to satisfaction with nursing services [25–30]. PTSD results in failure to present a favorable nursing services, causes diminished performance of nurses, and as it also leads to diminished nurses’ interest in their job [11, 31]. Considering the fact that no similar study has been performed in emergency departments of Iran, the aim of this study was to investigate the prevalence of PTSD and its related factors among nurses who worked in the emergency department.

## 2. Methods

In this cross-sectional study, all nursing personnel in the emergency unit of educational hospitals affiliated with Mazandaran University of medical sciences were evaluated in summer 2017. The total number of individuals employed in these units was 131. Data were collected using questionnaires. Identical questionnaires were used for all of emergency nurses. The questionnaire consisted of two parts. The first part included demographic characteristics such as age, educational level, marital status, working hours per month, direct contact with the patient, experience of traumatic accidents, and extent of interest in the job. The second part was Civilian Mississippi Scale for PTSD, which was utilized in this study to investigate the prevalence of PTSD. The mentioned questionnaires were distributed among emergency nurses and collected after completion. All of the nurses who had been working in the emergency unit of the hospitals for more than one month were included in the study. Absence of a traumatic event for the participant over the past six months (such as death of significant others and family members, accident by car, etc.) was another inclusion criteria. Junior nurses with less than one month of experience, no consent for participating in the study, and incomplete questionnaires were excluded from the study.

### 2.1. Civilian mississippi scale for PTSD

This questionnaire is a self-report scale developed by Keane *et al.* in 1988, which is utilized for evaluating the intensity of PTSD symptoms. This scale has 35 items, in which the participants respond to the items through a 5-point scale, with the items being scored as 1, 2, 3, 4, and 5. The total range of the scores of a person will be 35-175. Participant with score 107 and above representing presence of PTSD. The Cronbach alpha coefficient of this questionnaire has been reported to be 0.86-0.94. The validity and reliability of the Persian version of PTSD scale was obtained by Sadeghi *et al.* with desirable cohort evidence (r = 0.68, *p* = 0.001). Also, the validity coefficients of Cronbach alpha and index retest throughout the entire scale and its dimensions have been reported to be above 0.70 [32].

### 2.2. Ethical Considerations

The ethics committee of the university approved the protocol of the study. All the information related to this study was presented by the researcher both orally and in written form to the participants and written informed consent was obtained from them. Also, in order to help participants confide in the study, they were requested not to write their personal information and name on the questionnaires.

### 2.3. Statistical Analysis

Descriptive statistics were used for the demographic characteristics of nurses as well as descriptive variables. Data were analyzed using *Chi-square,* Pearson correlation coefficient, analysis of variance (ANOVA), Mann-Whitney and Kruskal-Wallis tests using SPSS Version 22. The *P* values less than 0.05 was considered statistically significant.

## 3. Results

In this study, all nursing personnel of the emergency unit of educational hospitals affiliated with Mazandaran University of medical sciences, who were 131 individuals, participated. Table 1 presents the demographic characteristics of the study population. Women constituted the major part of the study population (61.8%). A total of 111 individuals participating in the study (84.7%) had direct contact with patients. Also, 96 participants (73.3%) mentioned experience of traumatic accidents in their past personal experiences.

**Table 1:** Demographic characteristics of emergency nurses.

Demographic Information		Frequency	Percentage
yaer (age)	20-30	54	41.2
	31-40	48	36.6
	41-50	24	18.3
	51-60	5	3.8

Educational level	Bachelor	122	93.1
	Master	9	6.9

Marital status	Single	39	64.1
	Married	84	29.8
	Other	8	6.1

Gender	Male	50	38.2
	Female	81	61.8

Duration of employment	1-5 years	46	48.9
	5-10 years	36	27.5
	10-20 years	21	16
	Above 20 years	10	7.6

Working hours per month	Less than 100	12	12.2
	100-150	9	6.9
	150-200	60	45.8
	More than 200	46	35.1

Nurse to patient ratio	1:5	84	64.1
	1:8	27	20.6
	1:10	12	9.2
	1:15	6	4.6
	1:20	2	1.5

Direct contact with the patient	Yes	111	84.7
	No	20	15.3

Experience of traumatic events	Yes	96	73.3
	No	35	26.7

Interest in the job	Low	24	18.3
	Average	66	50.4
	High	41	31.3

Working shift	Night	62	47.3
	Day	69	52.7

Regular weekly exercise	Never	43	32.8
	Very low	37	28.2
	Low	15	11.5
	Average	22	16.8
	Regular	14	10.7
	Professional	0	0

In the present study, the total mean score of PTSD was 82.41 in emergency nurses. This value was 82.96 and 82.08% in men and women, respectively, which does not show any significant difference *(p* = 0.698). The mean score in nurses with a working background above 20 years was 75.07, while this value in those with a working background of less than five years was 87 (*p*-value = 0.659). In married nurses, the mean score was 85.33, which was not significantly different from 79.35 for the single nurses (p-value = 0.195). The mean of PTSD score, in terms of nurses’ demographic and professional characteristics have been shown in Table 2.

**Table 2:** Mean of PTSD score in terms of nurses’ demographic and professional characteristics.

Variables		No.	PTSD score (mean)	SD	*P*-value
Gender	Male	50	82.96	14.63	0.698
	Female	81	82.08	10.95	

Duration of employment	1-5 Years	64	87	9.57	0.659
	5-10 Years	36	82.69	15.07	
	10-20 Years	21	82	16.64	
	Above 20 Years	10	75.07	8.19	

Marital status	Married	84	85.33	13.44	0.195
	Single	39	79.35	10.54	
	Other	8	89.25	7.49	

Educational level	Bachelor’s	122	82.08	12.57	0.254
	Master’s	9	87	9.88	

Direct contact with the patient	With Direct Contact	96	78.02	13.32	0.545
	Without Direct Contact	35	83.51	9.69	

Experience of traumatic events	Yes	111	83	12.9	0.210
	No	20	79.20	8.72	

Interest in the job	Low	24	83	9.39	0.942
	Moderate	66	82.51	13.52	
	High	41	81.92	12.43	

Regular weekly exercise	Never	43	80.62	10.86	0.480
	Very Low	37	83.40	9.83	
	Low	15	79.40	20.96	
	Average	22	78.09	11.73	
	Regular	14	79.92	12.66	

Working shift	Night	62	82.20	13.13	0.855
	Day	69	82.60	11.87	

## 4. Discussion

In the present study, 82.96% of emergency nurses experienced PTSD, which is in line with the results of the studies by Laprosa and Alden in Canada [33] and Iranmanesh, *et al.* in Iran [34]. However, in the study by Mealer *et al.* in USA, only 18% of subjects showed PTSD symptoms [17]. This discrepancy can be due to the different target group in the present study, which investigated nurses currently employed in the emergency units of hospitals. However, in the study by Mealer *et al.* [17], PTSD was investigated among nurses of all units. The differences in the administrative structure of hospitals, as well as different sample size, sampling method, and level of honesty in responding to questions can be other reasons of this discrepancy. In this study, the prevalence of PTSD was higher in nurses with a shorter working background, though this difference was not statistically significant, which is consistent with the study by Laprosa and Alden [33]. This might be related to greater experience of the senior personnel in performing their tasks while keeping their working spirit, strengthening their defense mechanisms, or habituation to stresses. In addition, the range of accidents that are detrimental to a person decreases over time. Also, the mean score of PTSD was higher in married nurses, though this difference was not significant. Marital status can also predict PTSD; married individuals are more subject to this disorder, which was in line with the study by Narimani, *et al.* in Iran [35]. This finding can be justified with regards to stressful factors and more responsibilities of married individuals compared to their single counterparts, though this difference was not statistically significant.

Comparing the nurse to patient ratio, the groups 1:15 and 1:20 had the maximum percentage of PTSD, but this difference was not significant. This suggests that greater occupational pressure and the probability of confronting more stressful accidents in business can be introduced as a risk factor for PTSD development in these individuals. Comparing nurses with different levels of education, prevalence of PTSD was higher in nurses with lower educational levels, but this difference was not significant. In the study by Narimani, *et al.* [35], the level of education and prevalence of PTSD were not related to each other. This discrepancy can be due to further training of nurses with higher levels of education. In the present research, PTSD was more prevalent in men than women, which was in line with the study by Laprosa and Alden [33]. This difference was not statistically significant. The results of the study by Narimani, *et al*. showed an insignificant association between gender and PTSD symptoms among emergency nurses [35]. This increase of prevalence in men can be due to less expression of emotions. Having traumatic events in life experiences, indirect contact with the patient, and being not interested in the job have resulted in higher prevalence of PTSD in nurses. On the other hand, regular weekly physical activity has been effective in reducing PTSD. In spite of the difference in the working shifts of nurses in emergency units of hospitals, this difference did not result in development of difference in their level of stress. The results obtained from this study suggest that the nurses of emergency wards who dealt with more traumatized patients and emergency patients were more at risk of developing anxiety disorders including PTSD. Studies suggest that among all individuals who had met the diagnostic criteria for PTSD, around 98% suffered from burnout. Also, those with PTSD and occupational burnout had more problems in their personal life compared to those who only had burnout [17]. Since emergency units of hospitals is considered a stressful setting due to high workload, referral of traumatized patients, as well as burn and emergency patients, the faced-paced environment, greater psychological preparation of the emergency personnel is recommended so that they can do their tasks in the best way [36, 37].

Availability of social support and possibility of talking to colleagues are really important. Supportive preventive measures can be effective for preventing and treating PTSD among nurses in the emergency ward [21, 38, 39]. Caring for the psychological health of personnel, the managers of the healthcare area should consider systematic and dynamic educational programs for their adaptation in order to help this group of staff [40, 41]. Further, for better management of hospitals and enhancing their efficiency, a special care should be planned towards managing PTSD symptoms in employees, whereby changing the conditions of interpersonal communication can be considered as an important step to mitigate PTSD [10].

Nevertheless, development of PTSD in emergency nurses may be influenced by a wide variety of personal, social, and occupational factors [42], which should be further investigated in future studies. Also, additional studies are required to evaluate the efficacy of psychiatric consultation and also providing educational support packages in diminishing the PTSD symptoms among emergency nurses with PTSD.

The limitation of the present study was existence of confounding variables such as the baseline status of the person before employment and history of employment in other jobs and wards, which can affect the obtained results.

## 5. Conclusion

The results of this study revealed the high prevalence of PTSD among nurses who worked in emergency department. Therefore, it seems that designing and providing supportive and educational services to emergency nurses can be effective for preventing and managing this disorder, which probably can improve their performance.

## Author’s contributions

Study conception and design: SMH, FJ and FE. Acquisition of data: SMH, MSK, HM and SMP. Statistical analysis and interpretation of data: SMH and FJ. Drafting of the manuscript: SMH, FJ, FE, HM, MSK and SMP. Critical revision of the manuscript for important intellectual content: SMH, FJ, FE, HM, MSK and SMP.

## Conflicts of interest statement

The authors declare no conflicts of interest.

## Financial support

This study has been financially supported by the deputy of research and technology, Mazandaran University of Medical Sciences, Sari, Iran.
